# Mapping Health Literacy Research in the European Union: A Bibliometric Analysis

**DOI:** 10.1371/journal.pone.0002519

**Published:** 2008-06-25

**Authors:** Barbara K. Kondilis, Ismene J. Kiriaze, Anastasia P. Athanasoulia, Matthew E. Falagas

**Affiliations:** 1 Alfa Institute of Biomedical Sciences (AIBS), Athens, Greece; 2 Hellenic American University, Athens, Greece; 3 Department of Medicine, Tufts University School of Medicine, Boston, Massachusetts, United States of America; Canadian Agency for Drugs and Technologies in Health, Canada

## Abstract

**Background:**

To examine and compare the research productivity on selected fields related to health literacy of the current members of the European Union, the four candidate countries waiting to join the EU, Norway, Switzerland, and the United States.

**Methodology/Principal findings:**

A bibliometric analysis (1991–2005). Data sources included papers published by authors from each country separately. The 25 European countries produce less than 1/3 health literacy research when compared to the U.S. (13,710 and 49,523 articles were published by authors with main affiliation in the European Union and the four candidate countries, and the U.S., respectively). The Netherlands and Sweden (followed by Germany, Italy, and France) are the European countries with the highest number of research published in fields related to health literacy. After adjustment for population Sweden, Finland, and Norway, were on the top of the relevant list. In addition, Sweden, Finland, and Ireland, were on the top of the list of countries regarding research productivity on the selected fields after adjustment for gross domestic product (GDP).

**Conclusions/Significance:**

Inequalities in research published on the topic of health literacy exist among Europe, Norway, Switzerland, and the U.S. More research may need to be done in all areas of health literacy in Europe and the potential detrimental effects of this gap should be further investigated.

## Introduction

Health literacy is becoming an important focal point for health providers in many countries around the world. One predictor of health literacy is patient health outcomes. Health literacy is defined as “the degree to which individuals have the capacity to obtain, process, and understand basic health information and services needed to make appropriate health decisions” [1]. According to research, individuals with low health literacy are more likely to have poor health, are less likely to understand their health problems and treatment management, and are at higher risk of hospitalization [2], [3], [4].

U.S. readability studies indicate that text written for the general public needs to be developed with a goal to reach individuals between the 6^th^ and the 8^th^ grade reading level, in order to cover the majority of the population [5], [6]. Overall, older persons have lower functional health literacy, and lower literacy than younger people of successive generations who have continued a full-time educational course [5], [6], [7].

In 2001, the U.S. Center for Health Care Strategies estimated that low functional literacy resulted in an estimated $32 to $58 billion in additional health care costs [8]. These costs included additional hospital stays and office visits, longer hospital stays, extra tests, procedures, and prescription medications. The impact that health literacy has in the European countries has been difficult to measure as standardized tools such as readability formulas have been tested only in English, French, or Spanish [9], [10] and standardized tests for health literacy (TOFHLA, S-TOFHLA, REALM) have been tested only in English [4], [11] though other health literacy assessment tools have also been devised in Spanish [12]. Given that there has not yet been established a way to measure health literacy in most European countries, there is limited research published in this field regarding the impact of low health literacy to health care costs.

According to the European Opinion Research Group in 2003 [13], 41% of the original 15 EU surveyed countries believed that the Internet is a good source of health related information. Wilson et al, state “about half of the people seeking health information on the Internet believe that the Internet has a major impact on their understanding of health problems and on their interaction with their doctor” [14]. Therefore it is important that through the new development of information technology in the European countries, health related websites are credible, meeting quality criteria such as accountability, accessibility, and usability [13], [15].

According to a brief bibliometric analysis that was previously performed by our group, health literacy, readability, health competence and informed consent constitute research areas are considerably neglected in Europe, these areas in total being about 25% of the global research production in this field (defined in our previous paper as “health literacy” categories) [16]. The aim of this study is to quantify the published amount of health literacy research literature available in Europe by looking at the individual countries of the European Union (EU), the candidate countries, Norway, Switzerland, and the U.S. The estimation of productivity of these countries on health literacy may help identify specific countries that are deficient in this field by absolute and relative to other countries terms. Such data may sensitize public health officials to help boost the attention in the health literacy field.

## Methods

Our study covered the period of 1991 to 2005. We examined data for the U.S. plus the two groups of the European Union countries (the current twenty-five countries and the four “candidate” countries: Bulgaria, Croatia, Romania, Turkey), and two additional European countries: Norway and Switzerland. The U.S., Norway, and Switzerland were selected because of their high gross domestic product (GDP) devoted for research and development. We explored papers published by authors from each country in the selected fields that were included in the PubMed database. For the bibliometric analysis, we initially selected search terms that would best describe the different aspects of health literacy.

We performed several literature searches and focused our search on 13 fields: health perception, health literacy, readability, readability formulas, readability and health, health knowledge, health awareness, health and communication, health promotion, health promotion materials, health competence, informed consent, and health information. The results still included many published papers that were not of direct relevance to health literacy. Repeating the testing of the methodology for better accuracy, a few words were selected that could be excluded (if found in the content of the articles) in each field mentioned above, narrowing down our results and increasing the specificity. An example of the search term structure is: Readability NOT DNA, NOT “monkey” AND France [ad].

Parentheses and quotes could affect the search outcome. For example, the phrase “health and communication” in PubMed is recognized as an alias for the Journal of Health Communication. In order to bypass this, we searched for the phrase using parentheses. For the phrases “Health Knowledge”, “Health Information” and “Readability and Health” we used double quotes around the phrase keeping the terms combined rather than the database searching for each term in the selected phrase individually (we present a description of terms in the Appendix S1). Following the final selection of terms, in order to improve specificity, we performed random selection verification tests to examine the reliability of each term.

For the database to generate published work within each country and exclude articles that were written about that country by a foreign researcher, the search was limited to the address of the author matching the country selected. For example in searching the term *Health Literacy* in France we limited the search to country address (France [ad]). This enabled us to obtain estimates for those individual countries' research productivity in health literacy.

The total amount of research produced was estimated by each country separately and by each group using the results. In addition, we used the online World Bank database to retrieve the information on the average population size, the mean GDP, and percentage of gross domestic product devoted to research and development, in order to evaluate the adjusted for these variables research productivity in the selected fields; years covered were 1991 to 2004 (which was the last year for available data).

## Results

We identified 13,710 articles published by authors with main affiliation in the European Union and the four candidate countries, 751 published in Norway, 772 in Switzerland, and 49,523 articles published by U.S. authors. The random selection verification tests indicated that most articles fell above 85% accuracy for the terms defined (range of accuracy was 60%–100%). Among EU nations, the countries with the highest number of research publications in fields related to health literacy are the Netherlands, and Sweden, followed by Germany, Italy, and France. (Table 1)

**Table 1:** Number of publications on selected fields originating from different countries*.

	Health Perception	Health Literacy	Readability**	Health Knowledge	Health Awareness	Health & Communication	Health Promotion ***	Health Competence	Informed Consent	Health Information	TOTALS
**EU**											
**Austria**	29	11	5	32	22	62	19	6	57	10	253
**Belgium**	77	28	0	78	34	171	54	37	89	10	578
**Cyprus**	0	0	0	3	3	2	3	2	0	0	13
**Czech Republic**	6	8	0	6	5	23	16	2	5	5	76
**Denmark**	84	59	9	87	37	161	99	31	50	5	622
**Estonia**	4	0	0	5	2	9	6	3	1	1	31
**Finland**	65	143	15	142	51	193	157	111	49	11	937
**France**	141	61	40	145	84	300	92	52	255	41	1,211
**Germany**	149	69	48	132	88	418	124	90	312	26	1,456
**Greece**	23	20	4	40	29	101	34	17	28	4	300
**Hungary**	3	7	1	13	7	20	18	12	12	1	94
**Ireland**	60	25	9	99	59	131	88	60	35	21	587
**Italy**	149	86	41	128	104	287	88	52	292	20	1,247
**Latvia**	1	0	0	1	0	2	0	0	2	0	6
**Lithuania**	4	2	0	5	2	6	9	2	3	2	35
**Luxemburg**	0	0	0	0	0	0	0	0	0	1	1
**Malta**	1	0	0	6	3	7	2	2	0	3	24
**Netherlands**	256	176	37	246	85	585	203	182	233	21	2,024
**Poland**	19	17	1	37	14	28	38	9	15	2	180
**Portugal**	7	13	2	6	6	26	12	3	7	2	84
**Slovakia**	1	0	0	1	1	3	4	0	0	0	10
**Slovenia**	3	4	0	2	4	19	4	2	5	1	44
**Spain**	97	78	21	68	56	178	65	28	90	12	693
**Sweden**	273	156	28	339	164	505	217	218	98	22	2,020
**United Kingdom**	78	19	23	83	60	169	71	43	62	8	616
**EU-25**	1,530	982	284	1,704	920	3,406	1,423	864	1,700	229	13,142
**EU-CCs**											
**Bulgaria**	1	0	4	8	0	8	3	2	6	1	33
**Croatia**	12	6	4	14	6	22	5	4	6	3	82
**Romania**	1	3	0	1	1	12	3	1	1	2	25
**Turkey**	42	72	3	109	35	60	21	25	58	3	428
**EU-CCs**	56	81	11	132	42	102	32	32	71	9	568
**Totals (EU-25+EU-CCs)**	1,586	1,063	295	1,836	962	3,508	1,455	996	1,771	238	13,710
**Norway**	97	87	10	101	48	216	83	65	35	9	751
**Switzerland**	75	46	15	92	67	190	151	43	75	18	772
**United States**	3,616	2,928	808	7,452	2,800	14,379	6,872	4,637	4,822	1,209	49,523

* EU-CCs = Candidate countries to the European Union.

** Results for “Readability”, “Readability formulas”, and “Readability and health” have been added together and are presented in the Readability column.

*** Results for “Health promotion” and “Health promotion materials” are presented in the Health Promotion column.

The research productivity on the selected fields originating from various countries after adjustment for population, gross domestic product, and spending for research and development for each country is presented in Table 2. After adjustment for population Sweden, Finland, and Norway, were in the lead. In addition, Sweden, Finland, and Ireland, were on the top of the list of countries regarding research productivity on the selected fields after adjustment for GDP. The research productivity for the current 25 countries of the EU adjusted for population was 16% of the productivity of the US, adjusted for the same variable.

**Table 2:** Adjusted total of research productivity on selected fields originating from different countries.*

	Average population (million)*	Average GDP (billion constant 2000 US$)*	Proportion of GDP for R&D %	Average annual spending on R&D (billion constant 2000 US$)	Total papers (1991–2005)	Total papers per population in millions	Total papers per GDP (billion constant 2000 US$) during the study period	Total papers per spending on R&D (billion constant 2000 US$) during the study period
**Austria**	8	177.4	1.9	3.4	253	31.6	0.10	5.0
**Belgium**	10.2	212.1	2	4.2	578	56.7	0.18	9.1
**Cyprus**	0.7	8.3	0.2	0.0	13	18.6	0.10	52.2
**Czech Republic**	10.3	53.7	1.2	0.6	76	7.4	0.09	7.9
**Denmark**	5.3	147.1	2.2	3.2	622	117.4	0.28	12.8
**Estonia**	1.4	16.6	0.6	0.1	31	22.1	0.12	20.7
**Finland**	5.1	107.8	3.1	3.3	937	183.7	0.58	18.7
**France**	58.4	1,225.50	2.2	27.0	1,211	20.7	0.07	3.0
**Germany**	81.8	1,774.50	2.4	42.6	1,456	17.8	0.05	2.3
**Greece**	10.8	105.6	0.6	0.6	300	27.8	0.19	31.6
**Hungary**	10.2	43.2	0.8	0.3	94	9.2	0.15	18.1
**Ireland**	3.7	78.1	1.2	0.9	587	158.6	0.50	41.8
**Italy**	57.4	1,019.30	1.1	11.2	1,247	21.7	0.08	7.4
**Latvia**	2.5	7.5	0.4	0.0	6	2.4	0.05	13.3
**Lithuania**	3.6	11.7	0.6	0.1	35	9.7	0.20	33.2
**Luxemburg**	0.4	16.6	1.7	0.3	1	2.5	0.00	0.2
**Malta**	0.4	3.3			24	60.0	0.48	
**Netherlands**	15.7	335.5	2	6.7	2,024	128.9	0.40	20.1
**Poland**	38.5	146.6	0.7	1.0	180	4.7	0.08	11.7
**Portugal**	10.1	96.7	0.8	0.8	84	8.3	0.06	7.2
**Slovakia**	5.4	19	0.8	0.2	10	1.9	0.04	4.4
**Slovenia**	2	17.4	1.4	0.2	44	22.0	0.17	12.0
**Spain**	37.8	516.7	0.9	4.7	693	18.3	0.09	9.9
**Sweden**	8.8	220.2	3.8	8.4	2,020	229.5	0.61	16.1
**United Kingdom**	58.6	1,331.50	1.9	25.3	616	10.5	0.03	1.6
**EU-CCs**								
**Bulgaria**	8.2	12.9	0.5	0.1	33	4.0	0.17	34.1
**Croatia**	4.5	17.6	0.9	0.2	82	18.2	0.31	34.5
**Romania**	22.5	39.2	0.5	0.2	25	1.1	0.04	8.5
**Turkey**	64.6	181.3	0.6	1.1	428	6.6	0.16	26.2
**All EU Countries (EU 25+EU CCs)**	546.9	7,942.9	1.8	146.7	13,710	25.1	0.12	6.2
**EU-25**	447.1	7,691.9	1.9	145.2	13,142	29.4	0.11	6.0
**EU-CCs**	99.8	251.0	0.6	1.5	568	5.7	0.15	25.2
**Norway**	4.4	152.5	1.6	2.4	751	170.7	0.33	20.5
**Switzerland**	7.1	233.8	2.7	6.3	772	108.7	0.22	8.2
**United States**	274	8,833.3	2.6	229.7	49,523	180.7	0.37	14.4

**Abbreviations:** GDP = gross domestic product; R&D = research & development; EU-CCs = candidate countries (those waiting to join the European Union); EU-25 =  the 25 members of the European Union.

* GDP and population for 2005 has been calculated based on the previous year, due to unavailability of data for 2005.

## Discussion

The main finding of our study is that the current 25 European countries' production of health literacy articles is less than 1/3 of those produced in the United States. Also, there is considerable inequality regarding research productivity on the selected fields between European countries. Though the importance of health literacy, with respect to health behavior, motivation and health outcomes, has generally been well described in the medical and social science literature, little is known about the research production on health literacy in Europe. When comparing the EU to the U.S. in health literacy research productivity our results show that the EU falls considerably behind the U.S. in this academic field compared to the total research productivity [17], except to research production in some fields such as Microbiology and Parasitology where Western Europe ranked highest [18], [19].

Inequalities in research production existing among European nations may be due to language barriers, not publishing all research performed, and placing more emphasis on medical and biological sciences, rather than social sciences. One additional factor is that several countries in Central and Eastern Europe have more recently gone through political and economic transitions. As the health care system was divided into two main tiers, public and private, the public has been directly impacted in terms of the quality of services and type of health care they receive [20].

One recent editorial further highlights the concept of health literacy as extending beyond health professionals' understanding of their patients' health decision-making and improving health information delivery for better medical care, to a lay persons' understanding the terms “health literacy” [21]. A person's literacy level and the readability of material are critical components of health literacy.

Digital literacy, the ability to use Information and Communication Technologies, has become as important as “classic” literacy [22]. Since using technology for education has become an important component of health literacy we therefore included the terms *health information* in our search. In 2002 about 40% of EU households had their own Internet connection [23]. However, unequal access to information technology exists since four out of the five websites are in the English language [24]. Besides, not all websites in the English language are readable enough to be of practical use for potential readers [25]. It is important that through the new development of information technology in the European countries, health related websites are credible, meeting quality criteria such as accountability, accessibility, and usability [13], [15].

The importance of empowering citizens to make healthy choices and participate in their health care is a major aspect of health literacy and a key topic in many European conferences and presentations [26]. According to reports on new members of the European Union, citizen's participation in their healthcare appears only in the report produced in Slovenia [27]. Thus, the European public may indeed subscribe to the issue of low health literacy, which in turn becomes a major economic and political burden on societies. No precise cost figures related to health literacy for Europe are known. Providing health information and building health literacy will enable improvement in persons' overall health and possibly reduce health care costs and existing European budgets.

Improving health literacy should include the following: training health educators to use instructional theories and teaching methods to create culturally and linguistically appropriate health education materials; helping providers use specific teaching strategies during health visits with patients who have limited literacy; and creating a shame-free environment for patients [28]. The U.S. Institute of Medicine and the Agency for Healthcare Research and Quality further suggest that difficult language used by health care professionals creates communication barriers for patients in understanding their diagnosis, medication instructions, and recommendations to prevent disease [29]. This is particularly relevant to informed consent and patients questioning their health providers about their health status as well as treatment options.

Our study has several limitations. First, the methods we used included search only in the PubMed search engine and not in non-PubMed journals. Our methodology could not effectively deal with multiple publications and did not allow us to estimate the number/proportion of articles that had authors from various countries. The term health literacy and its components, may be described differently by European versus American authors due to language differences, political as well as cultural norms. A factor impacting the accessible information regarding health literacy in European countries is the native language of each respective country. As we reviewed the results of the initial phase, we found the need to adjust the search method for research developed in European countries, in order to capture the majority of the articles published in health literacy or related fields. Due to the fact that in Europe, the term health literacy is not as commonly used as it is in the United States, we selected alternate terms that were used in articles when referring to concepts of health literacy, in respect not only to the terminology which may vary from some European countries to others, but also the language and translation barriers in relevance to those terms. Countries publish much of their research in their native language and health literacy terms may not be the same as those used in the U.S.

Also, the PubMed database may limit some results in a bibliometric study since retrieval varies over time due to the continual addition to the database and the varying indexing terms. Selection bias may also be a factor considered in the methodology used for selecting health literacy terms. However, both the review of several studies defining health literacy and its components in designing this study's methods, and the selective random test performed to improve reliability may have reduced selection bias. We cannot infer from our analysis whether there is, and to what degree if any, publication bias in the field of health literacy.

Based on the selective random test and review of articles' titles and abstracts, there are several notable contexts of the health literacy terms. Health literacy articles used educational levels or “functional health literacy” not standardized health literacy tools, and some articles referred to the educational backgrounds of health care staff. Readability articles included readability formulas and how “readable” health information is. Health knowledge included patient and provider knowledge as well as a few sources of knowledge (i.e. library, statistical data). Health awareness articles ranged from specific topic information and increasing the public's “awareness”, to health care providers' attitudes towards patients and their treatment. Health promotion articles often focused on future promotional planning. Most health competency articles reflected health care providers' training. Finally, informed consent was typically mentioned in the articles and several studies focused on the former as a unique topic.

Our quantitative results do not imply that more is necessarily better, without assessing the quality of the research produced by the respective European regions. Thus, additional qualitative and quantitative analyses need to be done by each country focusing on the quality of these health literacy studies. In addition, the results of papers already published by countries should be compared with the country's health outcomes including disease rates, life spans, or infant mortality. Thus a country's health outcomes, positive or negative, should deem whether more health literacy research is needed.

**In conclusion,** the 25 European countries produce less than 1/3 research in health literacy when compared to the U.S. Inequalities in research published on the topic of health literacy exist among Europe, Norway, Switzerland, and the U.S. These inequalities may be explained by language barriers, unpublished research, and variation in the terms of health literacy. More research may need to be done in all areas of health literacy in Europe and standardized assessment tools such as readability formulas and tests of functional health literacy should be developed and tested in native languages.

## Supporting Information

Appendix S1 (0.03 MB DOC)
