# Supplementary material for: Mapping Health Literacy Research in the European Union: A Bibliometric Analysis
Source: PLoS One. 2008 Jun 25;3(6):e2519. doi: 10.1371/journal.pone.0002519 (PMC2424242; doi:10.1371/journal.pone.0002519)
Supplement: Appendix S1 — (0.03 MB DOC) [file pone.0002519.s001.doc]

##### APPENDIX

**Table A**. **Exact search terms and coded limits***

| **Exact term** | Words Excluded |  |  |
| --- | --- | --- | --- |
|  |  |  |  |
| **health perception** | NOT bodily sensation NOT noise NOT environmental risks NOT validity NOT hearing | AND | Country[ad] |
| **health literacy** |  | AND | Country[ad] |
| **readability** | NOT DNA NOT "monkey" | AND | Country[ad] |
| **readability formulas** |  | AND | Country[ad] |
| **(readability AND  health)** |  | AND | Country[ad] |
| **"health knowledge"** | NOT quality management NOT economics NOT health economics NOT regulation | AND | Country[ad] |
| **health awareness** | NOT quality management NOT economics NOT health economics NOT regulation | AND | Country[ad] |
| **(health AND  communication)** |  | AND | Country[ad] |
| **health promotion** |  | AND | Country[ad] |
| **health promotion  materials** |  | AND | Country[ad] |
| **health competence** | NOT management NOT quality control | AND | Country[ad] |
| **informed consent** |  | AND | Country[ad] |
| **"health information"** | NOT emergency department NOT insurance policies NOT pharmacoeconomics NOT ultrasound NOT radiography NOT devices NOT ergonomics NOT informatics NOT information security NOT down syndrome | AND | Country[ad] |
|  |  |  |  |
| * United States was coded as: (USA[ad] OR United States of America[ad]);  All terms/countries have date limits January 01, 1991 to December 31, 2005;  except for the term “readability” (no publication date limits). | | | |
